# High Sugar-Induced Insulin Resistance in *Drosophila* Relies on the Lipocalin *Neural Lazarillo*

**DOI:** 10.1371/journal.pone.0036583

**Published:** 2012-05-02

**Authors:** Matthieu Y. Pasco, Pierre Léopold

**Affiliations:** Institute of Biology Valrose (iBV), CNRS UMR 7707, INSERM UMR 1091, University of Nice-Sophia Antipolis, Nice, France; Michigan State University, United States of America

## Abstract

In multicellular organisms, insulin/IGF signaling (IIS) plays a central role in matching energy needs with uptake and storage, participating in functions as diverse as metabolic homeostasis, growth, reproduction and ageing. In mammals, this pleiotropy of action relies in part on a dichotomy of action of insulin, IGF-I and their respective membrane-bound receptors. In organisms with simpler IIS, this functional separation is questionable. In *Drosophila* IIS consists of several insulin-like peptides called Dilps, activating a unique membrane receptor and its downstream signaling cascade. During larval development, IIS is involved in metabolic homeostasis and growth. We have used feeding conditions (high sugar diet, HSD) that induce an important change in metabolic homeostasis to monitor possible effects on growth. Unexpectedly we observed that HSD-fed animals exhibited severe growth inhibition as a consequence of peripheral Dilp resistance. Dilp-resistant animals present several metabolic disorders similar to those observed in type II diabetes (T2D) patients. By exploring the molecular mechanisms involved in *Drosophila* Dilp resistance, we found a major role for the lipocalin Neural Lazarillo (NLaz), a target of JNK signaling. *NLaz* expression is strongly increased upon HSD and animals heterozygous for an *NLaz* null mutation are fully protected from HSD-induced Dilp resistance. NLaz is a secreted protein homologous to the Retinol-Binding Protein 4 involved in the onset of T2D in human and mice. These results indicate that insulin resistance shares common molecular mechanisms in flies and human and that *Drosophila* could emerge as a powerful genetic system to study some aspects of this complex syndrome.

## Introduction

Complex organisms living in changing environment need to adapt their energy supply to energy-costing processes such as metabolism, growth and reproduction. In many organisms, this adaptation relies on insulin/IGF signaling (IIS), as loss of components in IIS leads to important metabolic and growth defects. The dichotomy between fuel metabolism and growth control as seen in mammals is relying on the action of two distinct hormones, insulin and Insulin-like Growth Factor-I (IGF-I), exerting their cellular effects through the activation of distinct receptors. This is exemplified by the strikingly differences of the phenotypes observed upon genetically removing either the receptor for insulin (IR) or the receptor for IGF-I (IGFR-IR) [1], [2]. This functional separation is not fully clarified yet, and it is unclear whether it is due to an intrinsic capacity of each ligand/receptor to activate distinct intracellular pathways or to extrinsic differences such as the tissue distribution of each receptor [3]. Evolution-wise, this setup is restricted to the vertebrate phylum and most animal species make use of more primitive insulin/IGF systems (IIS), raising the issue of how independently these physiological regulations might be carried out in other phyla.

Invertebrates like *Drosophila* use a conserved IIS consisting of seven insulin-like peptides (ILP) called Dilps, expressed in diverse tissues, suggesting that they carry distinct functions [4]. A recent genetic analysis of single DILP mutants shows overlap as well as complementarities between the different DILP genes in controlling functions as diverse as growth, metabolism, stress resistance, reproduction and aging [5]. Remarkably, such functional diversity relies on the activation of a single membrane-bound receptor of the Receptor-Tyrosine-Kinase family called InR, activating a downstream cascade of unique effectors, consisting of an insulin-receptor substrate (Chico), a PI3K-PDK1-AKT module and a single forkhead-box O transcription factor (dFoxO). Loss-of-function studies for InR and its downstream cellular components indicate that the InR pathway controls the physiological functions carried out by the different Dilps. At the adult stage, this includes fuel metabolism, stress resistance, fertility and aging [6]. During early development, the function of IIS is restricted to the control of tissue growth and fuel metabolism. Since in flies a unique set of cellular components is used to respond to circulating insulin-like peptides, including InR and downstream components, any modulation of circulating Dilp levels is expected to impact on both functions.

We have previously observed that upon nutritional stress (deprivation of amino acid in the diet), Drosophila larvae experience growth inhibition largely due to a control of brain Dilps secretion, leading to a reduction in circulating Dilps [7]. This growth defect was accompanied by an elevation in circulating carbohydrate levels. This observation, contrasting with the reduced glycemia observed in rodents exposed to diet restriction or starvation, suggested that *Drosophila* larvae cannot separate fuel homeostasis from growth regulation. The Dilps are not the only metabolically active hormone in *Drosophila*. A glucagon-related hormone called Adipokinetic hormone (AKH) is also produced from the corpora cardiaca (CC) and participates in glucose homeostasis [8], [9]. More importantly, CC cells express the orthologs of Sur1 and Kir6.1, two subunits of an ATP-sensitive potassium channel that allows coupling circulating glucose levels and hormone secretion [8]. This ability is not shared with the brain insulin-producing cells (IPCs), which suggests that the main actor in carbohydrate homeostasis is AKH rather than the Dilps. Nevertheless, varying the levels of circulating Dilps strongly impacts glycemia, suggesting a functional obligation to couple fuel homeostasis and growth during larval stage.

Here we show that larvae fed a high sugar diet (HSD) accumulate high levels of circulating glucose and are strikingly smaller than control animals. This indicates that the unique *Drosophila* IIS cannot exert separate control on growth and metabolism upon changing environmental conditions. We further demonstrate that HSD-induced growth inhibition is due to resistance to insulin-like peptides in peripheral tissues. We finally uncover a conserved molecular mechanism for this process involving the production of the secreted lipocalin Neural Lazarillo (NLaz), an ortholog of the vertebrate Retinol Binding Protein 4 (RBP4) implicated in the onset of type II diabetes (T2D) in mice and human.

## Results

### High sugar diet (HSD) affects larval growth

**Figure 1:**
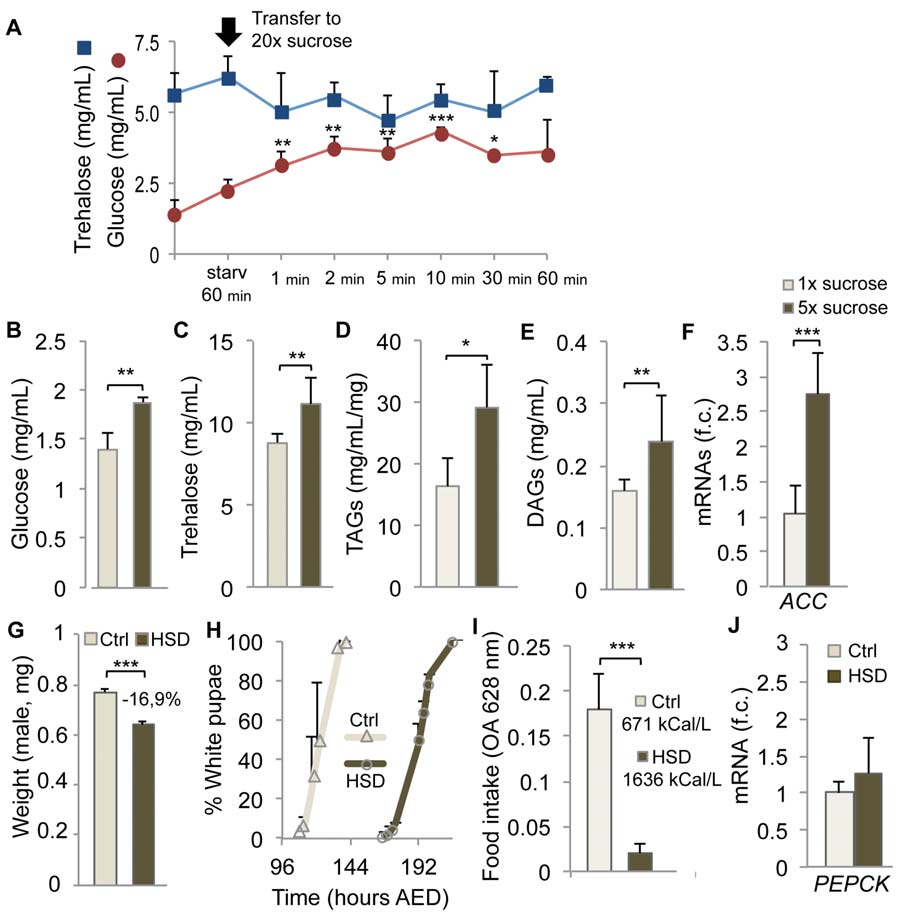
Metabolic and growth defects induced by High Sugar Diet. (A) After 60′ of starvation, L3 larvae were transferred on high sucrose (20× in PBS), and the levels of circulating glucose and trehalose were monitored from 1′ to 60′ after transfer, revealing a modification of glucose levels, but not trehalose. Note that the starvation before transfer to 20× sucrose induces itself a slight increase in basal glycemia. (B) Glucose and (C) trehalose levels as measured in the hemolymph of wandering larvae fed from eclosion on 1× or 5× sucrose diet. (D) Total triacylglycerides (TAGs) and (E) circulating DAGs in larvae fed on 1× and 5× sucrose diet after eclosion. (F) Effect of 1× or 5× sucrose diet on the rate of transcription of the *ACC* gene in mid-L3 larvae. (G) Weight of adult males emerged after larvae were fed on 1× (ctrl) or 5× sucrose diet (High Sugar Diet, HSD). (H) Effect of control diet or HSD on the developmental timing, assessed at the time of white pupa formation. (I) Measurement of food intake of L3 larvae previously fed on ctrl diet or HSD, as measured by blue food ingestion. (J) Differential expression of the *PECK* gene on ctrl diet or HSD in mid-L3 larvae.

Previous data [7] indicate that a reduction of circulating Dilp levels induced by low amino acid diet concomitantly impacts growth and carbohydrate metabolism. We envisaged testing this coupling by carrying experiments where larvae are exposed to conditions perturbing fuel homeostasis, and are checked for growth abnormalities. For this purpose, *Drosophila* larvae were raised on food with increased sucrose levels and a time course was realized to evaluate the kinetics of changes in circulating carbohydrates *in vivo*. Both glucose and the disaccharide trehalose are present in the larval hemolymph. Because of its non-reducing properties, trehalose can accumulate at higher concentration than glucose (6 mg/ml *vs* 1 mg/ml) without toxicity for the different tissues that bathe in the hemolymph. After switching larvae on a very high sucrose diet (1.2 g/ml sucrose in PBS, called 20×), we observed a rapid increase in circulating glucose levels stabilizing at 3–4 mg/ml after only 2 minutes (Figure 1A). Remarkably, trehalose levels did not increase within such a short time window, and did not seem to be affected even after 1 hr of treatment, possibly due to the fact that trehalose metabolism is controlled by a long term hormonal process [10]. This indicates that the immediate metabolic response to a high sugar diet is an increase in free hemolymph glucose. We then observed the effect of long term exposure to moderately high sugar and for this purpose, we fed larvae immediately after eclosion on either 1× (60 mg/ml, normal sucrose concentration in fly food) or 5x-sucrose concentrations in an otherwise normal food recipe consisting of yeast, cornmeal and agar. The 5× sucrose recipe (called hereafter High Sugar Diet or HSD) is rather syrupy and its sugar concentration compares with that of a hazelnut/chocolate spread recipe. Larvae fed HSD presented increases in both hemolymph glucose and trehalose when measured at wandering stage (Figure 1B and 1C). This was accompanied by an increase in total triacylglycerides (TAG) as well as diacylglycerides (DAG) circulating in the hemolymph (Figures 1D, E). In line with these results, the transcription of acetyl-coA carboxylase (ACC), a limiting enzyme for fatty acid synthesis, was strongly induced (Figure 1F). Therefore, *Drosophila* larvae show different metabolic adaptations to short and long term exposure to high sugar, with, in both cases a sensible variation in free circulating glucose levels. Furthermore, long-term metabolic adaptation to high sugar diet in *Drosophila* larvae resembles that of vertebrate with increased circulating carbohydrates and fat. For this reason, free circulating glucose rather than trehalose was taken as a marker of carbohydrate homeostasis in all our experiments.

HSD should logically induce an over-production/release of Dilps in the larval hemolymph to counteract increased glycemia. As a consequence, animals raised in these conditions would be expected to reach bigger size than control animals. Surprisingly, adult males born on HSD presented a strong reduction in mass (−16.9%), indicative of a reverse interference of HSD on growth control (Figure 1G). This growth deficit was accompanied with an important developmental delay (3 days, Figure 1H), consistent with systemic growth inhibition. A trivial explanation for such growth deficit would be that animals do not feed properly on HSD. We found that larvae raised on HSD showed reduced ingestion rate compared to animals raised on normal food (Figure 1I). However, HSD food contains 2.4-fold more calories per weight than normal diet and, due to the developmental delay, HSD-fed larvae feed for a longer period, suggesting that the total number of ingested calories on HSD compensates for the observed feeding defect. In line with this, the general level of transcription of the metabolic enzyme PEPCK commonly used as a marker of starvation [11]–[13] was not increased upon HSD feeding, indicating that, in these conditions, animals are not subjected to food deprivation (Figure 1J). This suggests that the growth deficit is not a consequence of feeding defect, but rather due to a modification of the machinery controlling tissue growth.

### HSD-induced growth inhibition is due to peripheral insulin resistance

**Figure 2:**
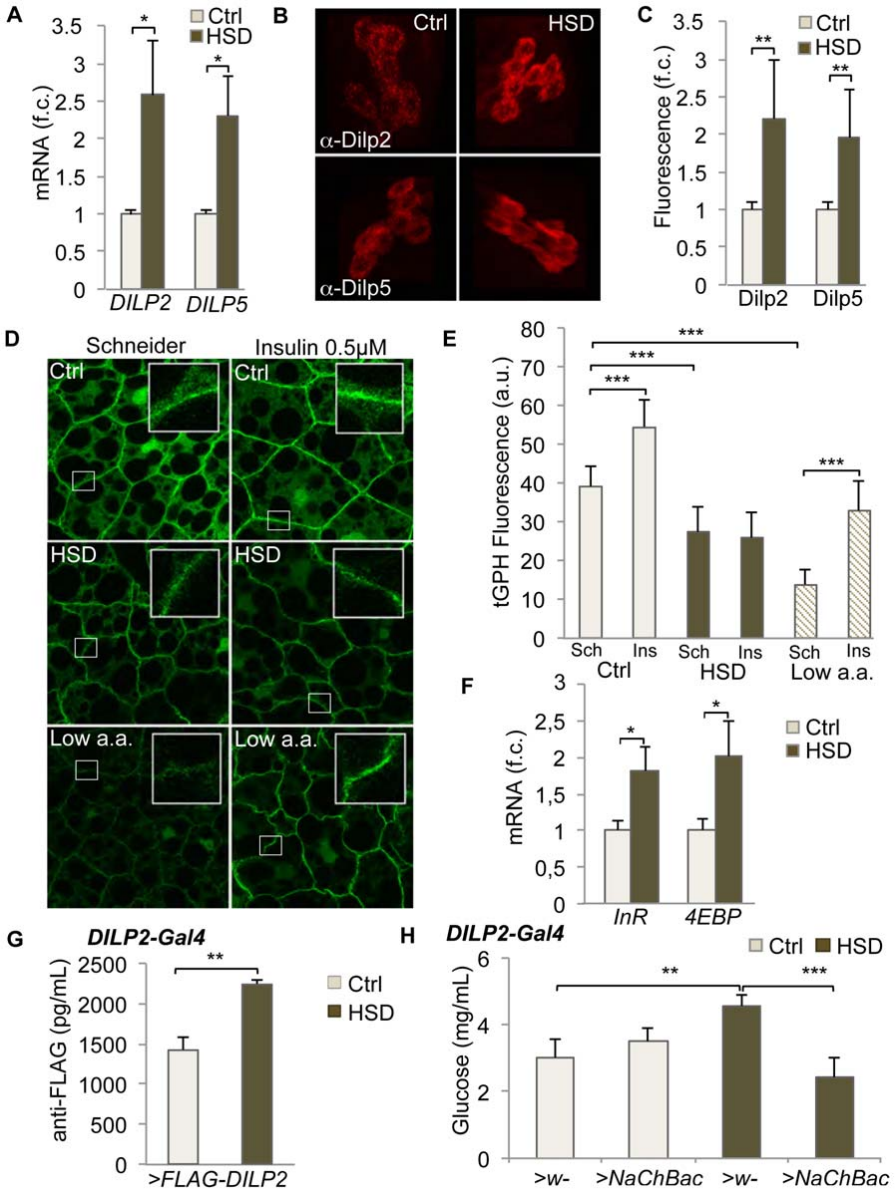
High Sugar Diet induces peripheral Dilp-resistance. (A) A two-fold increase in *DILP2* and *DILP5* transcription is observed in larval brain upon feeding a HSD (fold changes are presented, f.c.). (B) Dilp2 and Dilp5 immuno-staining of the insulin-producing cells (IPCs) in L3 larvae fed ctrl and HSD. (C) Quantification of fluorescence in IPCs (fold changes are presented, f.c.). (D) and (E) Insulin stimulation test of fat body explants from control or HSD-fed larvae. After a short incubation to human insulin (0,5 µM, 20 min) the amount of tGPH fluorescence was quantified as an evaluation of insulin sensitivity. In D, representative images of fat bodies after incubation. Cell membranes outlined with the tGPH marker are shown in inserts. (F) The dFoxo targets *Inr* and *4EBP* are upregulated in HSD conditions, indicative of a general reduction of IIS in HSD-fed larvae (mid-L3 larval samples, fold changes are presented, f.c.). (G) Circulating Dilp2-Flag in the hemolymph of larvae fed either control of HSD. Larvae express a Flag-tagged Dilp2 in the IPCs (*Dilp2-Gal4>Flag-Dilp2*) and circulating levels of Dilp2-Flag are quantified using an Elisa method (see Material and Methods). (H) Forced expression of a bacterial sodium channel in the brain IPCs during larval development (*Dilp2-Gal4>NaChBac,* see (6)) promotes Dilps secretion and prevents HSD-induced hyperglycemia.

We therefore examined expression and production levels of the different Dilp genes in the larval brain since brain Dilps have been recognized as a major source of growth inducers during larval development [14], [15]. We focused our analysis on Dilp2 and Dilp5, for which we can easily follow brain accumulation using specific antibodies [7]. Larvae raised on HSD showed a two-fold increase in *DILP2* and *DILP5* expression as measured by qRT-PCR analysis on dissected brains (Figure 2A). This was accompanied by a two-fold increase in Dilp peptide accumulation (Figure 2B and 2C), therefore suggesting a general increase in brain Dilp production upon HSD. We then tested whether HSD-fed animals presented elevated circulating Dilp levels. For this, animals expressing a Flag-tagged Dilp2 under the control of the *DILP2* promoter (*DILP2>DILP2-Flag*) were used and the level of circulating Dilp2-flag was determined by an Elisa assay using anti-Flag antibodies (see material and methods). In these conditions, we observed a 1.55 fold increase in circulating Dilp2-Flag upon HSD feeding, indicating that HSD-fed larvae are hyper-insulinemic (Figure 2G). An increase in insulin production and secretion associated with an increased glycemia is a characteristic of insulin-resistance. Therefore we tested whether HSD-raised animals could experience such resistance, which would explain a small size despite elevated circulating Dilp levels. For this purpose, we dissected fat body explants and kept them in *ex-vivo* culture either with or without added human insulin. In order to quantify the responsiveness of the tissue to insulin, we used a cell fluorescent marker called tGPH, allowing quantification of the activity levels of PI3K in living cells by measuring membrane-associated GFP fluorescence (see material and methods). Fat body explants dissected from larvae raised on normal medium showed increased membrane-associated GFP signal upon insulin treatment, indicating that they respond to insulin and activate the signaling cascade downstream of InR, leading to PI3K activation. In contrast, fat bodies from HSD larvae showed reduced basal levels of tGPH that did not increase upon insulin addition (Figure 2D and 2E). This indicates that after exposure to HSD, fat body cells have a reduced capacity to activate the signaling cascade downstream of InR, and have therefore become insulin resistant. As a control, fat bodies dissected from under-nourished larvae also presented reduced basal levels of tGPH fluorescence but retained a strong capacity to activate IIS in response to insulin (Figure 2D and 2E). A similar obliteration of insulin stimulation was observed in salivary glands from HSD fed larvae (Figure S1). Consistent with these results, general expression of *InR* and *4EBP* was markedly up-regulated in animals fed with HSD (Figure 2F). These genes are direct targets of dFoxO, a transcription factor inhibited by IIS. Therefore, an increase in *InR* or *4EBP* expression is a sign of IIS reduction. Finally, forced secretion of brain Dilps concomitant to HSD feeding was sufficient to prevent hyperglycemia, indicating that, as in the case of treatment of type II diabetes (T2D) by insulin secretagogues, promoting insulin release from insulin-producing cells can circumvent peripheral insulin resistance (Figure 2H).

These results indicate that the growth deficit observed in HSD is caused by a general reduction of IIS, itself a consequence of Dilp resistance in peripheral tissues.

### HSD-induced insulin resistance relies on the induction of the lipocalin NLaz

**Figure 3:**
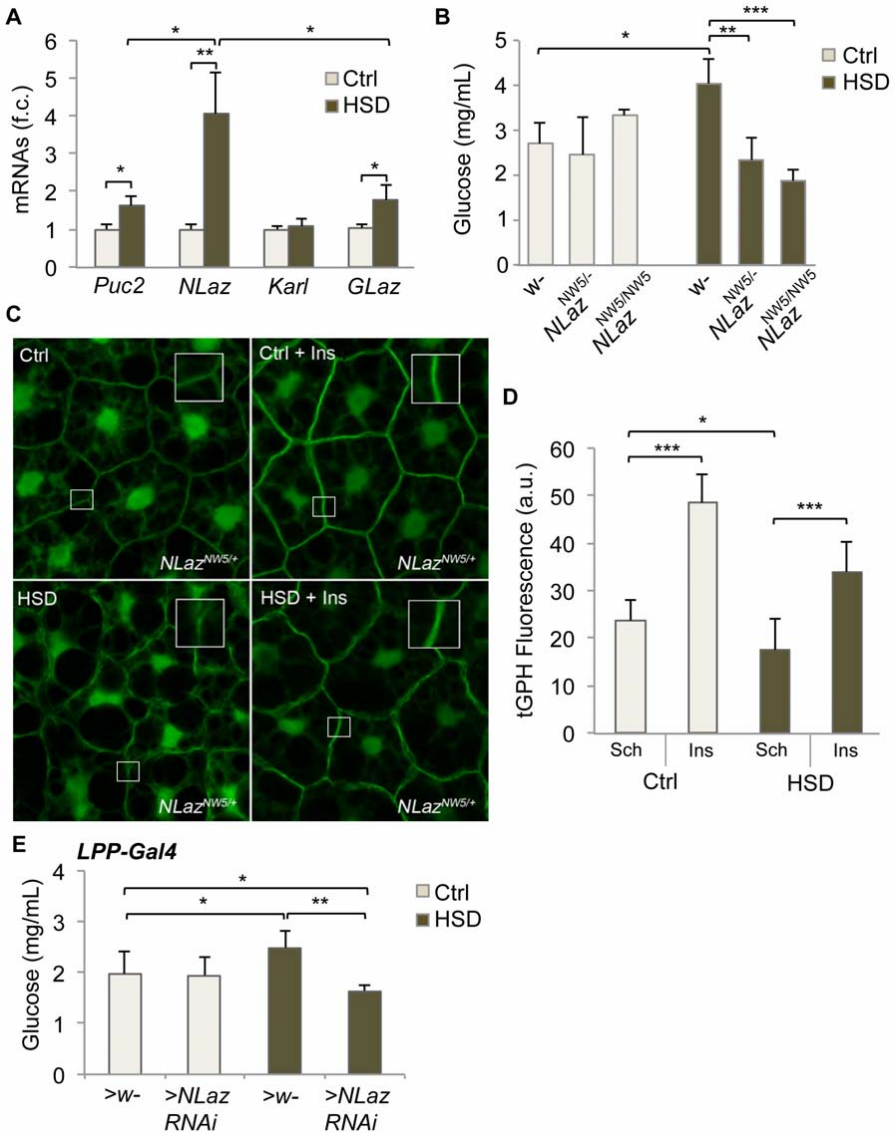
NLaz is required for High Sugar Diet-induced Dilp-resistance. (A) Changes in expression of *Puc*, *NLaz*, *Karl* and *GLaz* in HSD *vs* control conditions (fold changes are presented, f.c.). (B) Glycemia of control, heterozygous and homozygous *NLaz* mutant larvae fed either normal diet (light grey bars) or HSD (dark bars). (C) and (D) Fat body explants from *NLaz* mutant larvae fed either control or HSD were exposed to human insulin (0,5 µM, 20 min.). The amount of tGPH fluorescence was quantified as an evaluation of insulin sensitivity. (E) Glycemia of control larvae or larvae with a fat body-specific knock-down of *NLaz* (*NLaz-RNAi*), fed either normal diet (light grey bars) or HSD (dark bars).

Dilp resistance in HSD-fed flies presents obvious parallels with insulin resistance associated with T2D in obese patients. Several mis-regulations have been proposed to participate in insulin resistance in mammals. One common mechanism emerging is the activation of a cellular stress response, which in many systems, including *Drosophila*, opposes the activity of IIS through activation of the Jun-N-terminal kinase (JNK) pathway. We therefore explored the role of JNK activation in HSD-induced insulin resistance. First, HSD-fed larvae present an up-regulation of *puc*, a downstream target of the JNK signaling pathway (Figure 3A). Importantly, a direct target of JNK signaling, the Lipocalin-encoding *Neural Lazarillo* (*NLaz*) gene, is also induced by HSD (Figure 3A). NLaz is an ortholog of the vertebrate lipocalins Retinol Binding Protein 4 (RBP4) and Lipocalin 2. These molecules modify the sensitivity of peripheral tissues to insulin and their implication in controlling metabolic homeostasis is suggested in mammals and in flies [16]–[21]. We therefore evaluated a possible function for NLaz in HSD-induced insulin resistance. NLaz, Karl and GLaz are three members of the lipocalin family in *Drosophila* but only *NLaz* shows dramatic induction in HSD fed larvae, while *GLaz* is only moderately induced and *Karl* expression is not modified (Figure 3A). We then tested the capacity of an *NLaz* mutation to rescue the metabolic defects induced in HSD fed larvae. Heterozygous and homozygous *NLaz* mutants present normal glycemia when raised on normal diet ([21] and Figure 3B). By contrast, HSD-fed *NLaz^NW5/+^* or *NLaz^NW5/NW5^* larvae did not present elevated glucose levels, as observed in HSD-fed *wt* animals (Figure 3B). This suggested that even a partial reduction of *NLaz* function is sufficient to protect HSD-fed animals from insulin resistance. To test this directly, we dissected fat body explants from heterozygous *NLaz^NW5/+^* animals and subjected them to an insulin stimulation test. *NLaz^NW5/+^* fat body explants showed strong, indistinguishable response to insulin whether animals were fed a normal or a HSD diet (Figure 3C and 3D). This indicates that partial reduction of *NLaz* function is sufficient to fully restore peripheral insulin sensitivity in HSD fed larvae. Since *NLaz* is highly induced in the larval fat body in response to stress and JNK activation [21], we next tested whether reducing *NLaz* expression in fat cells would be sufficient to protect larvae from insulin resistance. Indeed, silencing *NLaz* expression specifically in the fat body rescued normal glycemia in animals fed on HSD (Figure 3E).

Therefore, through its activation in fat body cells, *NLaz* appears as a major player in the onset of high sugar-induced insulin resistance in flies.

## Discussion

### *Drosophila* IIS does not exert separate controls on metabolism and growth

One particularity of the insect IIS is the presence of a unique receptor for multiple insulin-like peptides. This raises the possibility that the multiple functions assigned to IIS might not be independently regulated following an acute variation in environmental conditions (the “coupling hypothesis”). We have tested this experimentally during larval development, where IIS controls both systemic growth and carbohydrate homeostasis. Our previous results showed that a limitation in dietary amino acids reduces circulating Dilps, which impacts both growth and carbohydrate homeostasis [7]. Here, we have used experimental conditions where carbohydrate metabolism is challenged by a high sugar diet and its effect on growth is monitored. HSD induced an increase in glycemia followed by increased insulinemia (high Dilp expression and accumulation in the IPCs, elevated Dilp2 concentrations in the hemolymph), which we anticipated to induce overgrowth. In contrast, HSD fed larvae gave rise to small flies due to Dilp resistance in peripheral tissue. This indicates that Dilp resistance in flies impacts both metabolic and growth functions. This raises the possibility that Dilps and IIS are not used to maintain glucose homeostasis in normal physiological conditions. Previous work has demonstrated that the fly glucagon AKH has a selective action on carbohydrate and lipid homeostasis without influencing growth [8]. Therefore, using AKH and not Dilps to control energy homeostasis would prevent larvae from accidental coupling between metabolism and growth. This possibility finds support in the fact that AKH cells, but not Dilp cells, couple secretion to variations in glucose and internal ATP levels [8]. In our experiments, we did not find noticeable changes in AKH expression or accumulation in the AKH-producing cells in response to HSD (data not shown). Moreover, there is strong experimental evidence that, in addition to their growth-promoting function, circulating Dilps can influence metabolic homeostasis [14], [15], [22]. This overall indicates that despite a conservation of its multiple functional outputs, the hard wiring of IIS in *Drosophila* does not allow a clear discrimination of growth and metabolic regulations during larval development. What are the respective contributions of Dilps and AKH to energy homeostasis in the adult fly are questions awaiting further investigation.

### Dilp resistance in flies parallels insulin resistance in mammals

In human studies, the link between dietary carbohydrates and the development of insulin resistance and type II diabetes has long been elusive, mainly because of the difficulty to evaluate glycemic loads and indexes from food questionnaires [23]. An increasing number of epidemiological studies now point to a role of carbohydrates in the emergence of T2D in human [24]–[26]. Here in less than four days of feeding on HSD, larval tissues become strongly resistant to the effect of Dilps *in vivo* and to human insulin *ex-vivo*. This insulin-resistant state is characterized by: (i) high glycemia despite increased insulinemia, (ii) increased lipid storage and circulating lipids, (iii) rescue by forced Dilps secretion, (iv) lack of response of peripheral tissues to stimulation by exogenous insulin. This last point was tested in different larval tissues including the fat body, which carries both hepatic and adipose functions in the larva. HSD-fed animals accumulate high lipid levels in the fat body, which becomes resistant to the action of exogenous insulin. This is reminiscent of metabolic alterations seen in response to over-nutrition in mammals, where lipid metabolites accumulate in the liver leading to liver steatosis, a hallmark of insulin resistance and T2D [27], [28]. In line with this, we find that *ACC* expression is strongly increased in the fat body of HSD-fed larvae. This enzyme transforms acetyl-CoA into malonyl-CoA, a precursor for lipogenesis and an inhibitor of CPT-1, which imports long chain acyl CoA in the mitochondria for beta-oxydation. Suppression of ACC2 activity in mice induces beta-oxidation and was shown sufficient to reverse hepatic insulin resistance [29]–[31]. Therefore, the fat body of HSD-fed animals is subjected to metabolic alterations similar to those taking place in the fatty liver of T2D or obese patients. These observations parallel those of Musselman and colleagues, who recently published a state of sugar-induced insulin resistance in *Drosophila* [32].

### NLaz/RBP4: a conserved actor of insulin resistance in mammals and insects

One striking finding is the fact that heterozygous *NLaz/+* animals are fully protected of insulin resistance when exposed to a HSD. NLaz is a *Drosophila* lipocalin that is strongly up-regulated upon HSD feeding. NLaz was previously shown to act downstream of JNK to maintain metabolic homeostasis, in part by controlling lipid biogenesis and circulating carbohydrate levels [21]. *NLaz* expression in the larval fat body reduces general IIS levels, whereas *NLaz* mutant larvae present elevated IIS [21]. We also find here that silencing *NLaz* in fat cells protects larvae from HSD-induced Dilp resistance. The role of NLaz as a potential adipokine antagonizing IIS for metabolic regulation is remarkably similar to the role of its mammalian orthologs, Lipocalin 2 and the Retinol-Binding Protein 4 (RBP4). Serum concentration of both lipocalins correlate with obesity, T2D and insulin resistance in human and mice, although some of these associations have been disputed in human patients in the case of RBP4 [16]–[20], [33]–[35]. The reduction of RBP4 concentration in diet-induced obese mice was shown to improve insulin sensitivity whereas injection of recombinant RBP4 decreases insulin sensitivity in normal mice, a phenotype associated with a strong induction of the neoglucogenic enzyme PEPCK [16]. In addition, a functional polymorphism in the RBP4 gene associated with increased serum RBP4 was found in a Mongolian population suffering rapid increase of diabetes [36]. These observations are functionally related to our present findings in *Drosophila* showing that heterozygosity for *NLaz* is sufficient to protect animals from diet-induced insulin resistance. In addition, the level of expression of the *Drosophila PEPCK* gene is strongly reduced in *Nlaz* mutant animals, even if ectopic expression of *Nlaz* is not sufficient to drive *PEPCK* expression (a result in line with the absence of *PEPCK* induction upon HSD) (Supplemental Figure S2 and Figure 1J). These data collectively suggest a common molecular basis for the mechanism of insulin resistance in organisms as distant as insects and mammals. Further work using both vertebrate and invertebrate models should help understand the role of circulating lipocalins in reducing insulin sensitivity in peripheral tissues.

In summary, our present study recapitulates in a highly genetically amenable system some of the interactions observed between genetic factors and environmental factors leading to T2D as pinpointed by epidemiological studies in patients. This is the demonstration that the fly can be used to screen for genes that predispose to insulin resistance with conserved functions in mammals. The clinical progression towards TD2 is still not well understood and the use of genetic models might prove useful to decipher some of its underlying mechanisms.

## Materials and Methods

### Fly Strains and Food

The following fly lines were used: *w^1118^; DILP2-Gal4* [15], [22], *Lpp-Gal4* [37], *UAS-FLAG-DILP2* [38], and *UAS-NaChBac* (Bloomington Stock Center), W118, tGPH [39], *UAS-Nlaz-RNAi* (VDRC KK line *#101321*, no off target gene, 95% extinction of *NLaz* expression using an *act-Gal4* driver on larval extract), *Nlaz^NW5^* [21].

In all experiments, animals were fed at 25°C. Fly food was prepared as followed 10 g/L agar, 34 g/L yeast, 82,5 g/L polenta, and 60 g/L sucrose for 1× sucrose medium, and 300 g/L for 5× sucrose medium. All experiments were performed from synchronised L1 larvae on test conditions. Calculation of calories in the food: polenta 3,57 kCal/g, yeast 4 kCal/g, sucrose 4,02 kCal/g, i.e. 671.2 Kcal/L for 1× sucrose diet versus 1636 kcal/L for 5× sucrose diet (HSD). Animals raised on HSD develop over 7days compared to 4 days on 1× diet.

### Sucrose tolerance treatment, circulating carbohydrates or glycerides measurements, and triacylglycerides measurement

L3 feeding larvae were washed and starved in PBS for 60′ and subsequently soaked in a sucrose 20× solution (0.8 g/mL sucrose in PBS). Hemolymph was collected from 10 larvae at different time points and diluted (1∶10) in homogenization buffer (5 mM Tris [pH 6,6], 2,7 mM KCl, 137 mM NaCl) and heated for 5 min at 70°C. First, glucose was measured after a 15 min incubation at 37°C using the Thermo Glucose GOD-POD assay kit (Thermo Fisher Scientific; Waltham, MA). Trehalose was converted with porcine trehalase (Sigma, T8778) overnight at 37°C and the total amount of glucose was measured the same way. Circulating carbohydrates and glycerides were measured from hemolymph collected from wandering larvae. For triglycerides measurement, 5 wandering larvae were flash frozen and then homogenized in PBS buffer (Tween 0.05%, Roche protease inhibitors). DAGs and TAGs were measured using the Thermo Triglycerides assay kit (Thermo Fisher Scientific; Waltham, MA). The amount of triglycerides per larvae was normalized to amino acids. Quantification of metabolites was performed using a Sunrise spectrophotometer plate reader at 510 nm for carbohydrates and triglycerides and 540 nm for amino acids. (Tecan; Mannedorf, Switzerland).

### Developmental delay

L1 larvae were collected 24 hr after egg deposition (AED, 4 hr egg collections) and reared at 30 animals/tube. The percent of white pupae was estimated from an average of 3 tubes per condition and each experiment was repeated four times.

### Weighing flies

L1 larvae were collected 24 hr after egg deposition (AED, 4 hr egg collections) and reared at 30 animals/tube. Groups of 10 adult males were weighed with a XP26 Deltarange microbalance (Mettler-Toledo; Greifensee, Switzerland).

### Food intake

Early L3 feeding larvae were transferred to fresh dye food (0.05% Brilliant Blue) for 10 minutes. After feeding, larvae were washed 3× in distilled water, dried and homogenized in 200 µL lysis buffer (50 mM Tris [pH 8], 150 mM NaCl, 0.5% NP40, 1 mM EGTA). After centrifugation for 5 min in microfuge, 1 µL of supernatant was analyzed in a spectrophotometer at 628 nm (Eppendorf; Hamburg, Germany). Triplicate measurements on three distinct experiments were conducted.

### Quantitative RT-PCR

Larvae were collected 74 hr after egg laying and were flash-frozen. Total RNA was extracted using QIAGEN RNeasy Lipid Tissue Mini Kit according to the manufacturer's protocol. RNA samples (5 µg per reaction) were reverse transcribed using SuperScript II (Invitrogen), and the generated cDNA was used for real-time RT-PCR (ABI Prism 7000 system, qPCR Mastermix Plus for SYBRGreen I, Eurogentec France; Angers, France), using 2.8 ng of cDNA template and a primer concentration of 300 nM. Rp49 was used as a normalizer. Four separate samples were collected from each condition and triplicate measurements were conducted. Primers were designed using the Primer Express software (Applied Biosystems; Foster City, CA).

### Immunostaining, insulin stimulation test and fluorescence quantification

Brains were dissected from larvae in PBS, fixed in PBS containing 4% formaldehyde for 20 min at room temperature, and extensively washed in PBS containing 0.3% Triton X-100 (PBT). Tissues were then blocked for 2 hr in PBT containing 5% BSA. Primary antibodies were incubated overnight at 4°C, and secondary antibodies for 2 hr at room temperature. Fat bodies were dissected from L3 feeding larvae on ice. The explants were rinsed twice in PBS and incubated with Schneider medium w/o human insulin 0.5 µM for 20 min at room temperature. Then, preparations were washed quickly and fixed in PBS+formaldehyde. Tissues were mounted in Vectashield mounting medium (Vector Laboratories, Inc.; Peterborough, UK), and fluorescence images were acquired using a Zeiss LSM 510 META confocal laser-scanning microscope. For brain staining, the antibodies used were: rat anti-Dilp2, rabbit anti-DILP5 (Géminard et al. 2009), anti-rat Alexa 546, and anti-rabbit Alexa 546 conjugated (1/500; Molecular Probes; Paisley, UK). To quantify Dilp2 levels, confocal Z series of the IPCs were obtained using a 1 µm step size and identical laser power and scan settings. Fiji software was used to generate sum-intensity 3D projections of the Z stacks (12 bit scanned images) and to measure total fluorescent intensity across the IPCs. Fat bodies of feeding L3 larvae were imaged using the same confocal microscopy and average fluorescence was measured in 20 random squared areas (16×16 pixels or 4×4 micrometers), each covering part of the plasma membrane in different cells.

### DILP2-FLAG Elisa assay

Hemolymph was collected from fed mid-third-instar larvae and diluted (1∶10) in Ringer buffer and heated 5 min at 70°C. Samples were processed with the Reversal Phase SpinTips protocol according to manufacturer instructions (C18 SpinTips sample Prep Kit, Protea Biosciences; Morgantown,USA). Samples were diluted (1∶4) in coating buffer and processed to ELISA assay in 96 well-microtiter plates (Immulon 4HBX, Thermo Scientific; Rochester, USA). Primary antibody was incubated overnight at 4°C (anti-FLAG, rabbit, 1∶5000, Invitrogen; Carlsbad, CA) and secondary biotinylated antibody (anti-rabbit, goat, 1∶1000, Thermo Scientific; Rochester, USA) for 1 hr at room temperature. Streptavidin poly-HRP (1∶20000, Thermo Scientific; Rochester, USA) was used for antibody detection together with TMB solution (Ultra TMB-ELISA, Thermo Scientific; Rochester, USA). Signal quantification was performed using a Sunrise spectrophotometer plater reader at 450 nm (Tecan; Mannedorf, Switzerland) and with the help of a dilution range of FLAG peptide (Flag peptide, Inivtrogene; Carlsbad, CA).

### Statistics

For all experiments, error bars represent SEM, and P values are the results of a Student's test provided by Microsoft Excel.

## Supporting Information

Figure S1 Response of salivary gland explants (A) and tGPH quantification (B) from control or HSD fed larvae to human insulin (0.5 µM). The amount of tGPH fluorescence was quantified as an evaluation of insulin sensitivity (a.u., arbitrary unit).
(TIF)

Figure S2 Changes in expression of *PEPCK* in *NLaz^NW5^/NLaz^NW5^*(A) or *da>NLaz* (B) L3 larvae *vs* control animals (fold changes are presented, f.c.).
(TIF)
