# Supplementary figures and images for: High Sugar-Induced Insulin Resistance in Drosophila Relies on the Lipocalin Neural Lazarillo
Source: PLoS One. 2012 May 2;7(5):e36583. doi: 10.1371/journal.pone.0036583 (PMC3342234; doi:10.1371/journal.pone.0036583)

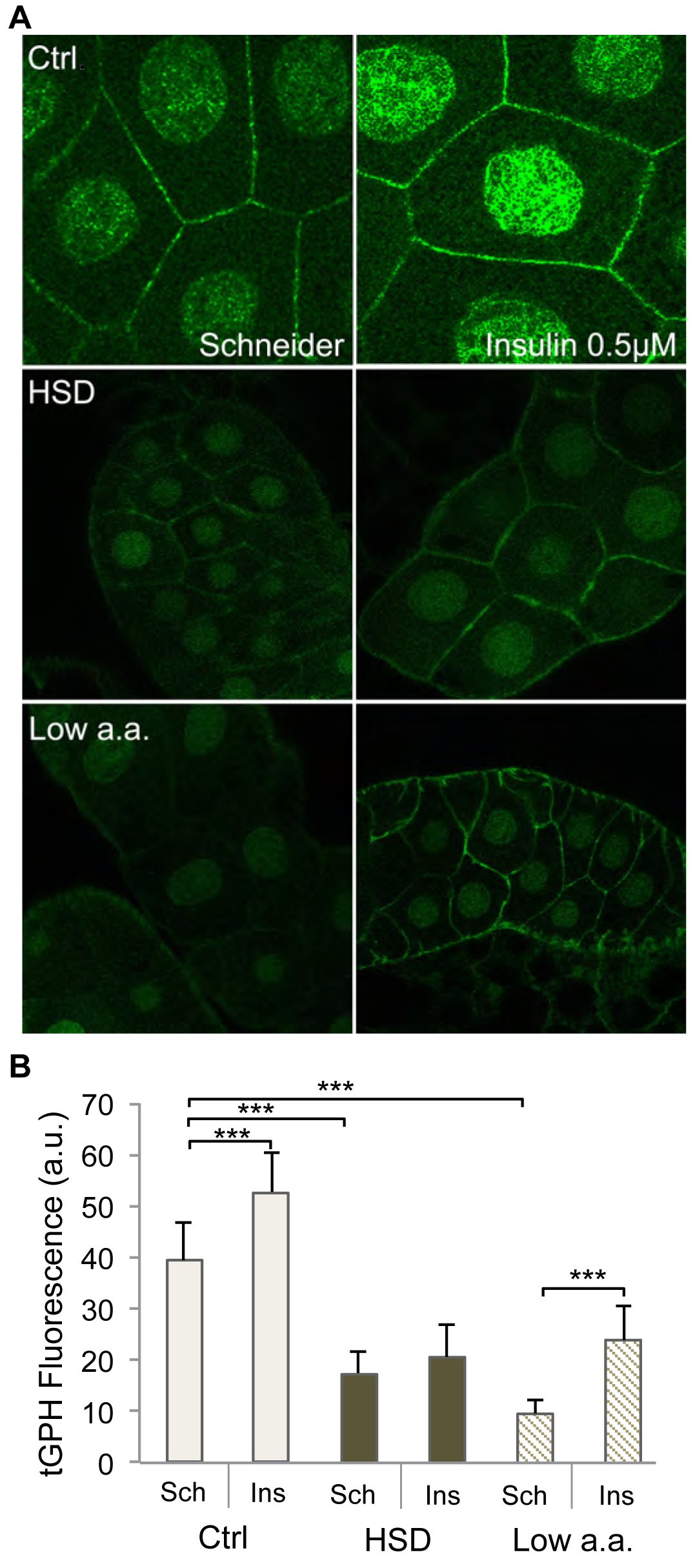

Supplement: Figure S1 — Response of salivary gland explants (A) and tGPH quantification (B) from control or HSD fed larvae to human insulin (0.5 µM). The amount of tGPH fluorescence was quantified as an evaluation of insulin sensitivity (a.u., arbitrary unit). (TIF) [file pone.0036583.s001.tif]

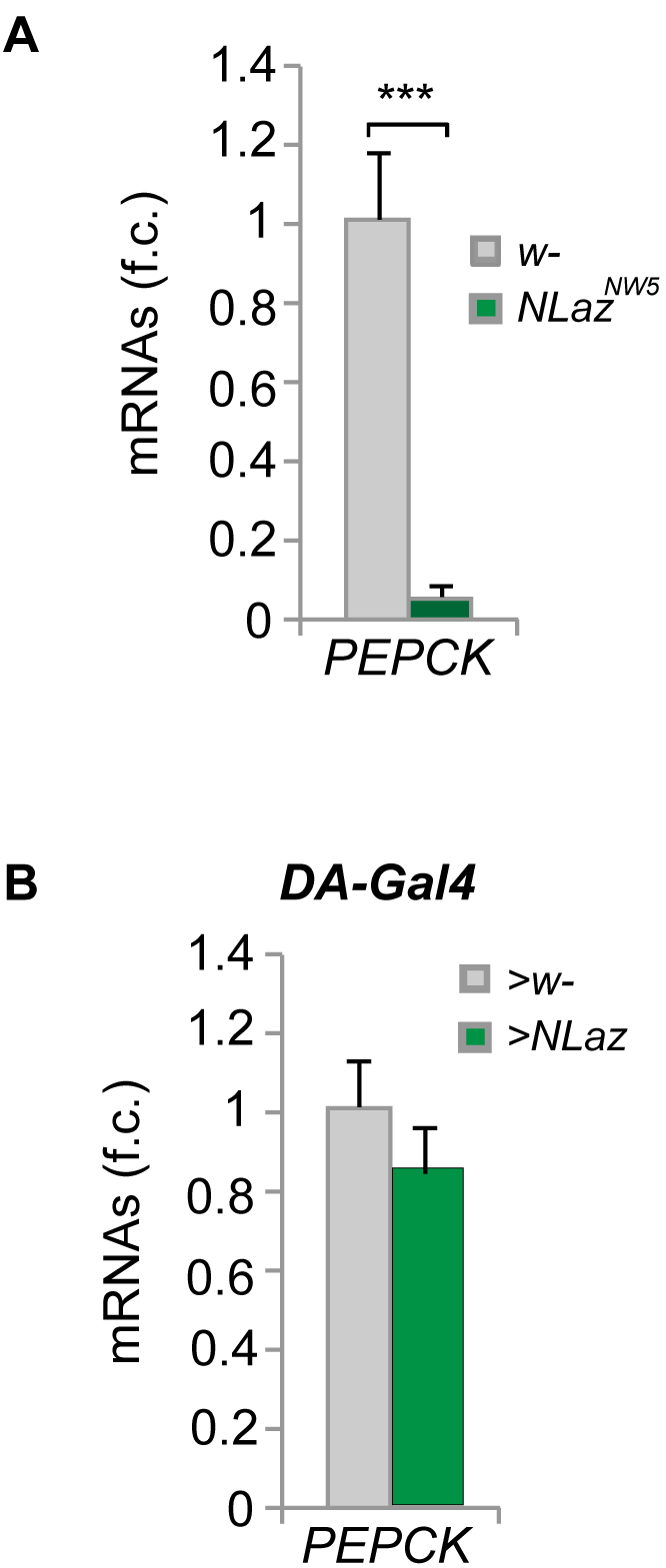

Supplement: Figure S2 — Changes in expression of PEPCK in NLazNW5/NLazNW5(A) or da>NLaz (B) L3 larvae vs control animals (fold changes are presented, f.c.). (TIF) [file pone.0036583.s002.tif]
